# MCPIP1 reduces HBV-RNA by targeting its epsilon structure

**DOI:** 10.1038/s41598-020-77166-z

**Published:** 2020-11-27

**Authors:** Yingfang Li, Lusheng Que, Kento Fukano, Miki Koura, Kouichi Kitamura, Xin Zheng, Takanobu Kato, Hussein Hassan Aly, Koichi Watashi, Senko Tsukuda, Hideki Aizaki, Noriyuki Watanabe, Yuko Sato, Tadaki Suzuki, Hiroshi I. Suzuki, Kazuyoshi Hosomichi, Makoto Kurachi, Kousho Wakae, Masamichi Muramatsu

**Affiliations:** 1 Department of Molecular Genetics, Graduate School of Medical Science, Kanazawa University, Kanazawa, Ishikawa 920-8640 Japan; 2 Department of Virology II, National Institute of Infectious Diseases, 1-23-1 Toyama, Shinjuku-ku, Tokyo, 162-8640 Japan; 3 Department of Pathology, National Institute of Infectious Diseases, 1-23-1 Toyama, Shinjuku-ku, Tokyo, 162-8640 Japan; 4 David H. Koch Institute for Integrative Cancer Research, Massachusetts Institute of Technology, Cambridge, MA 02139 USA; 5 Division of Molecular Oncology, Center for Neurological Diseases and Cancer, Nagoya University Graduate School of Medicine, Nagoya, 466-8550 Japan; 6 Department of Bioinformatics and Genomics, Graduate School of Advanced Preventive Medical Sciences, Kanazawa University, Kanazawa, Ishikawa 920-8640 Japan

**Keywords:** Hepatitis, Viral infection

## Abstract

Hepatitis B virus (HBV) is the major causative factor of chronic viral hepatitis, liver cirrhosis, and hepatocellular carcinoma. We previously demonstrated that a proinflammatory cytokine IL-1β reduced the level of HBV RNA. However, the mechanism underlying IL-1β-mediated viral RNA reduction remains incompletely understood. In this study, we report that immune regulator Monocyte chemotactic protein-1-induced protein 1 (MCPIP1) can reduce HBV RNA in hepatocytes. MCPIP1 expression level was higher in the liver tissue of HBV-infected patients and mice. Overexpression of MCPIP1 decreased HBV RNA, whereas ablating MCPIP1 in vitro enhanced HBV production. The domains responsible for RNase activity or oligomerization, were required for MCPIP1-mediated viral RNA reduction. The epsilon structure of HBV RNA was important for its antiviral activity and cleaved by MCPIP1 in the cell-free system. Lastly, knocking out MCPIP1 attenuated the anti-HBV effect of IL-1β, suggesting that MCPIP1 is required for IL-1β-mediated HBV RNA reduction. Overall, these results suggest that MCPIP1 may be involved in the antiviral effect downstream of IL-1β.

## Introduction

Hepatitis B virus (HBV), an enveloped DNA virus that relies on reverse transcription for replication and its persistent infection increases the risk of hepatocellular carcinoma^1^. Reverse-transcriptase inhibitors are used in anti-HBV treatments and efficiently suppress viral replication in hepatocytes with little side effect. However, they cannot completely eradicate the virus from the patients and emergence of drug resistant virus warrants investigation of novel targets for antiviral therapy.

Monocyte chemotactic protein-1-induced protein 1(MCPIP1), also known as ZC3H12A or Regnase-1, belongs to a CCCH-type zinc-finger family of four proteins (MCPIP1-4 or ZC3H12A-D). MCPIP1 contains the ubiquitin-associated (UBA) domain, *Nedd4*-BP1 and bacterial *YacP* Nuclease (NYN) domain, an RNA-binding CCCH-type zinc-finger domain, and an MCPIP1-unique proline-rich domain (PRD)^2,3^. Emerging evidence indicated that MCPIP1 promoted mRNA decay by recognizing a stem-loop structure in the 3′UTRs of several genes involved in inflammatory responses^4^. MCPIP1 preferentially recognizes the specific sequence, UAU and UGU, rather than ACA, AAA, and UCU, in the stem-loop structure^5^.

MCPIP1 reportedly inhibits various viruses, including human immunodeficiency virus (HIV)-1, hepatitis C virus (HCV), Japanese encephalitis virus (JEV), dengue fever virus (DEN), influenza A virus, Sindbis virus, and adenovirus^6–8^. Regarding HBV, the 5′ and 3′ ends of its pregenomic RNA (pgRNA) contain two epsilon structures, comprising 60 nt bulged stem-loops with UGU loop sequence. Therefore, we hypothesized that MCPIP1 targeted the epsilon stem-loop structure of viral RNA and investigated the role of MCPIPs in HBV infection. We found that MCPIP1 reduces HBV RNA, most likely by cleaving epsilon structure of pgRNA, and acts downstream of IL-1β-mediated antiviral pathway.

## Results

### MCPIP1 reduces HBV RNA

First we determined the correlation between MCPIP expression and HBV infection in the HBV-infected liver, by mining three public datasets. First, we found that the expression of MCPIP1, MCPIP2, and MCPIP4, but not that of MCPIP3, was increased in the liver tissue taken from patients with chronic HBV infection (n = 122), compared to healthy patients (n = 6) (Fig. S1A), based on the expression profiling of chronic hepatitis B (CHB) liver (GSE83148)^9^. Second, using the GSE52752 dataset, we found that MCPIP1 and MCPIP4 expression was significantly elevated (2.8 and 1.2 fold) in the livers collected from HBV-infected human liver-chimeric mice (8 weeks after infection) (n = 9), compared to mice in the control group (n = 6) (Fig. S1B). Third, MCPIP1 and MCPIP2 mRNA were increased in the primary human hepatocytes (PHHs) infected with HBV (GSE69590, Fig. S1C)^10^.

**Figure 1:**
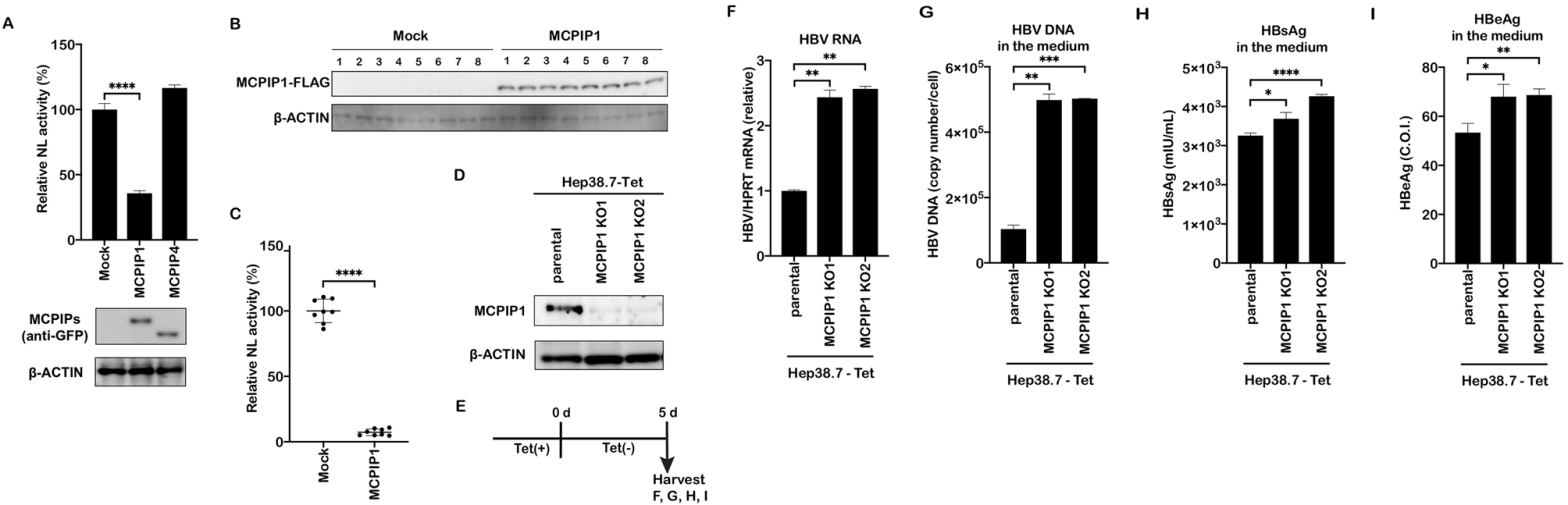
MCPIP1 reduces HBV RNA. (**A**) Huh7 cells were transfected with pgRNA reporter (pCMV1.2xHBV/NL), helper plasmid (pcDNA-CP), and pCAG-SEAP, together with GFP or GFP-MCPIP expression vectors. Cells were harvested 3 days after transfection. The total lysates were immunoblotted with anti-GFP or β-actin antibody. Nanoluc (NL) activity was normalized with SEAP activity, and the value of GFP (mock) was defined as 100%. (**B**,**C**) C57BL/6 mice were intravenously administered the pgRNA reporter, the helper plasmid pcDNA-CPtds, pCAG-SEAP, and FLAG-MCPIP1 or mock vector (n = 8 each). Two days after injection, lysates from the livers were subjected to Western blotting analysis (**B**) and luciferase assay (**C**). NL activity is indicated after normalization by SEAP activity. (**D**–**I**) Parental or MCPIP1 knockout Hep38.7-Tet cells were cultured in the absence of tetracycline for 5 days (**E**). (**D**) Validation of MCPIP1 ablation by Western blotting analysis. (**F**) RT-qPCR analysis to determine HBV RNA level, normalized by HPRT. The result is indicated as the ratio to the parental cells. (**G**) Supernatant HBV DNA qPCR analysis. The result is indicated by the absolute copy numbers. (**H**, **I**) The HBsAg (**H**) and HBeAg (**I**) levels in the supernatant were measured by chemiluminescent enzyme immunoassay. Expressed as international units mIU/ml and Cut-Off-Index (C.O.I.), respectively. *P < 0.05, **P < 0.01, ***P < 0.001, ****P < 0.0001.

To further investigate the role of MCPIP family in HBV infection, the HBV pgRNA reporter system was adopted^11,12^. In this system, a reporter gene nano-luciferase (NL) is inserted in the core region of HBV genome, and its activity reflects the HBV RNA level in the transfected cells. MCPIP expression vectors, HBV pgRNA reporter plasmid (pCMV1.2xHBV/NL), a helper plasmid (pcDNA-CP), and pCAG-SEAP (to monitor transfection efficiency) were cotransfected into Huh7 cells, followed by western blot and luciferase assay. We found that expression of MCPIP1, but not MCPIP4, significantly decreased the NL reporter activity (Fig. 1A). MCPIP2 and MCPIP3 were poorly expressed in this experimental condition (Fig. S2). In addition, hydrodynamic injection, an in vivo model to reproduce HBV replication in mice, was utilized^13^. C57BL/6 mice were injected with the pgRNA reporter and the helper plasmid pcDNA-CPtds, along with the MCPIP1 or mock vector. After 2 days, liver samples were harvested and the NL activity was determined. The NL reporter activity was lower in the livers injected with MCPIP1 vector, compared to the mock-injected livers (Fig. 1B,C).

Further to verify whether endogenous MCPIP1 contributes to viral restriction, MCPIP1 was knocked out by CRISPR/Cas9-mediated genome editing in Hep38.7-Tet cells (Fig. 1D), in which HBV replication starts from the chromosomally integrated HBV genome after the removal of tetracycline^14^. When we depleted tetracycline for 5 days, we found that the levels of HBV RNA, supernatant viral DNA, and HBsAg/HBeAg were higher in the knockout cells, compared with the parental (Fig. 1E–I). When tetracycline was added back to the cells after HBV accumulation (Fig. S3A), the viral RNA decreased in a significantly slower manner in both of the MCPIP1 knockout cells relative to the parental cells (Fig. S3B). Taken together, these results suggested that endogenous MCPIP1 also decreased HBV RNA level.

### Domains responsible for RNase activity and oligomerization of MCPIP1 are required for its anti-HBV activity

**Figure 2:**
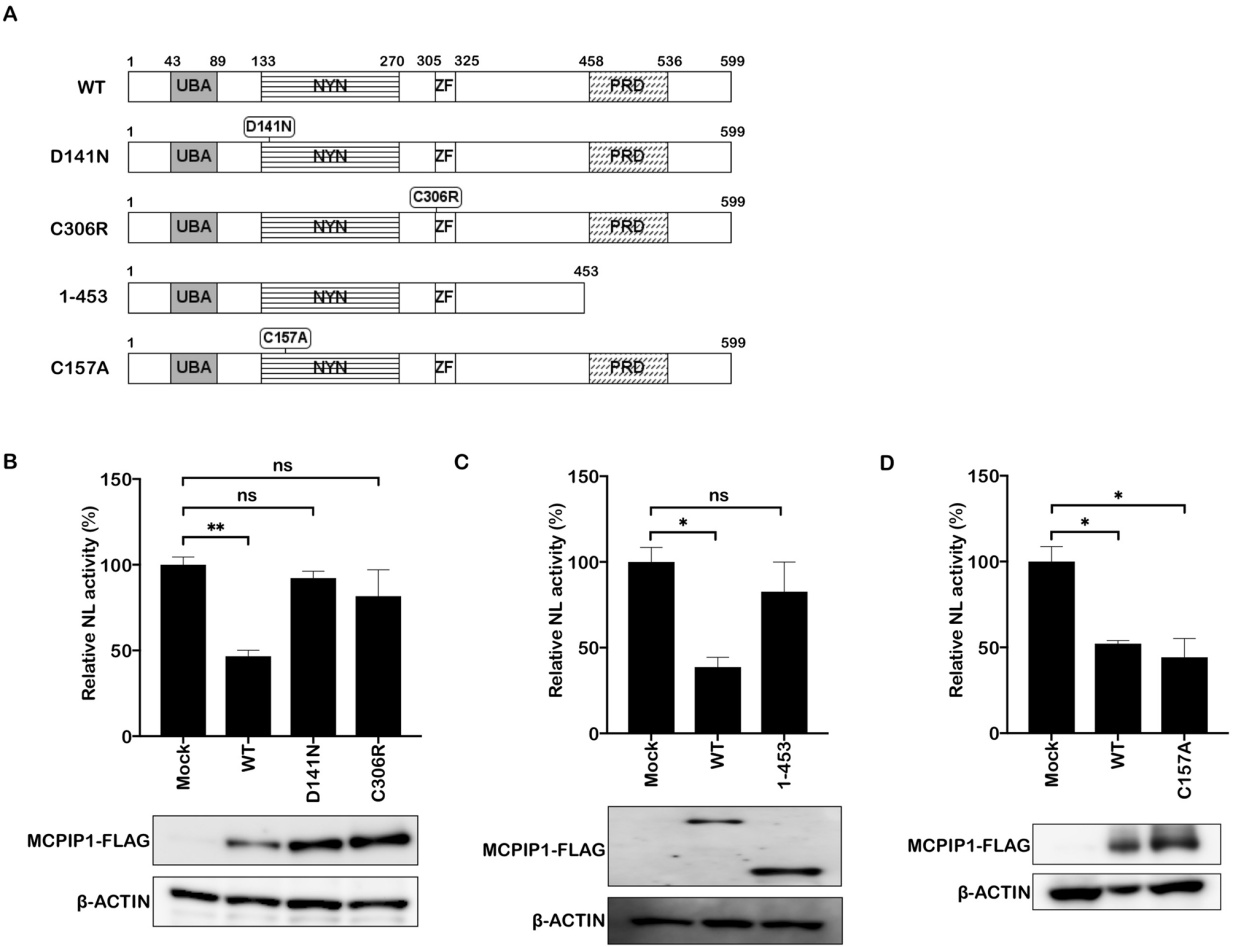
Domains responsible for RNase activity and oligomerization of MCPIP1 are required for its anti-HBV activity. (**A**) Scheme of MCPIP1 protein domains and MCPIP1 mutants used in the experiments. UBA, ubiquitin-associated domain; NYN, *Nedd4*-BP1*,* bacterial *YacP N*uclease domain; ZF, CCCH-type zinc-finger domain; PRD, proline-rich domain. (**B**–**D**) Huh7 cells were transfected with mock or FLAG-MCPIP1 expression vectors, including wild type (WT), and mutants (D141N, C306R, 1–453, and C157A), along with the pCMV1.2xHBV/NL, pcDNA-CP, and pCAG-SEAP. 3 days after transfection, cells were harvested and subjected to luciferase assay and Western blotting. NL activity of the mock is indicated as 100%. *P < 0.05, **P < 0.01.

The MCPIP1 protein has multiple domains (Fig. 2A)^2^, including an UBA domain (43–89) important for deubiquitination^3^; a NYN domain (133–270) and a CCCH-type zinc-finger domain (305–325), which are critical for its RNase activity; and a proline-rich domain (458–536), which triggers oligomerization^15^. To determine the contributions of these domains to the antiviral activity, we cotransfected the HBV pgRNA reporter vector with expression vectors encoding wild type or mutant MCPIP1 (Fig. 2A). The NL activity was lower in the cells overexpressing wild type MCPIP1, compared with that detected in the mock transfectants. In contrast, overexpression of D141N or C306R mutants, each harboring a single amino acid mutation in the RNase domain or CCCH-type zinc-finger, respectively^15^, did not result in decreased NL activity (Fig. 2B). Furthermore, a C-terminus truncated mutant (1–453) failed to decrease antiviral activity, which was comparable to that of the wild type (Fig. 2C). The D141N mutant is reportedly defective for both deubiquitinase (DUB) and RNase activity^16^. We further examined the anti-HBV activity of the C157A mutant, defective for DUB but not for RNase activity^16^, to determine the contribution of each activity in detail (Fig. 2D). We found that it lowered NL activity to a comparable level to that of the wild type, suggesting that RNase, but not DUB activity is important for the anti-HBV activity. Taken together, these results highlighted the importance of domains responsible for RNase activity, RNA-binding, and oligomerization, for the antiviral activity of MCPIP1.

### Epsilon structure of HBV RNA is required for MCPIP1-mediated reduction

**Figure 3:**
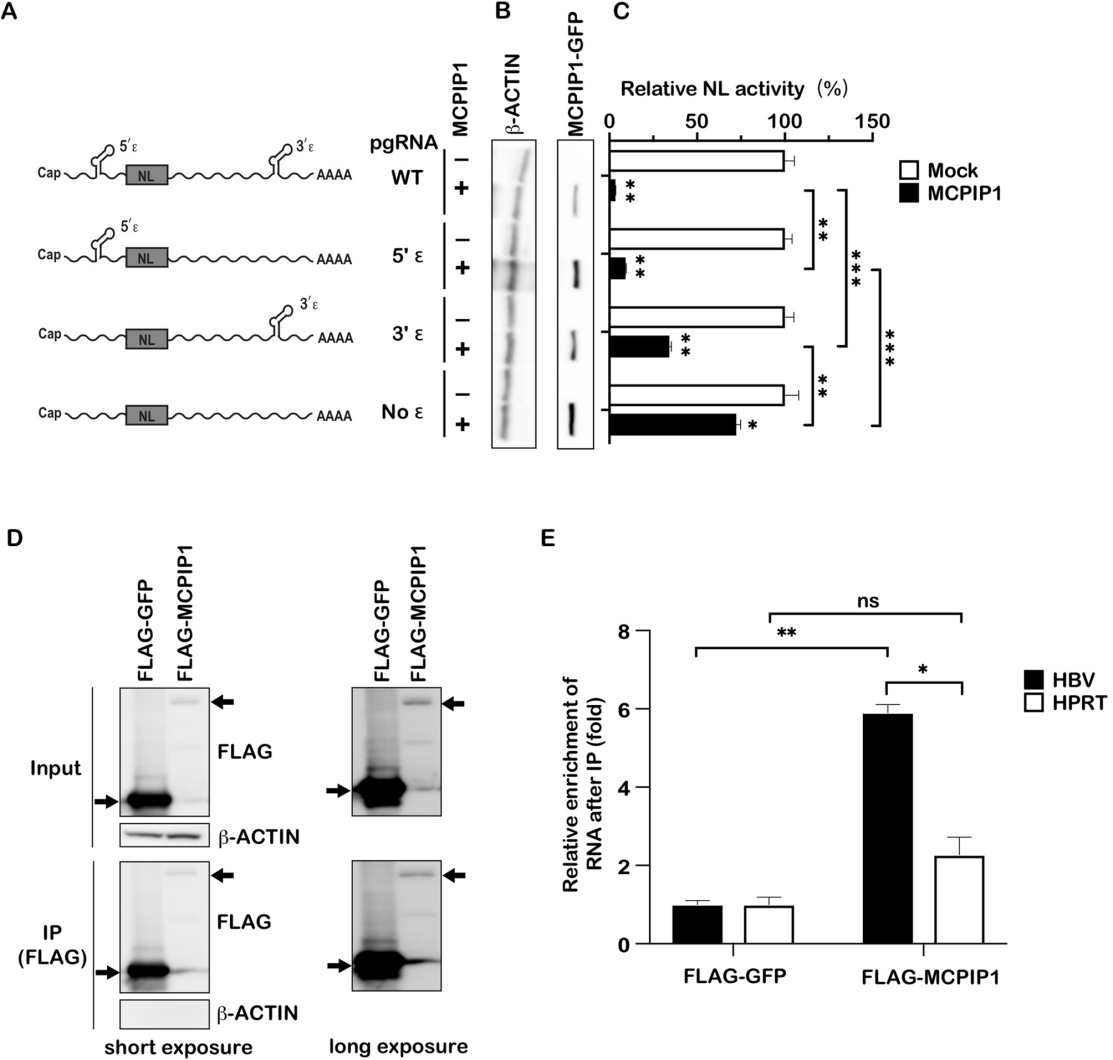
Epsilon structure of HBV RNA is required for MCPIP1-mediated reduction. (**A**) 293FT cells were transfected with pCMV1.2xHBV/NL, whose 3′- and/or 5′- epsilon structures are intact or deficient, along with the pCAG-SEAP, pcDNA-CP, and pGFP-MCPIP1. (**B**,**C**) 3 days after transfection, cells were harvested and subjected to Western blotting (**B**) and luciferase assay (**C**). In (**C**), NL activity of the cells transfected with pCMV1.2xHBV/NL and the mock plasmid was normalized to 100%. (**D**,**E**) Huh7 cells were cotransfected with pCMV1.2xHBV/NL, pCAG-SEAP, pcDNA-CP, and FLAG-MCPIP1 (or the mock plasmid, pFLAG-GFP). Transfected cells were further cultured for 3 days. Cell lysates were subjected to RNA immunoprecipitation assay using the FLAG M2 affinity gel. Crude extracts (input) and IP fractions were analyzed by Western blotting (**D**). The RNA (before and after immunoprecipitation) was subsequently subjected to RT-qRCR for quantitative evaluation of HBV RNA and HPRT mRNA. Relative enrichment of RNA in mock transfectants is defined as 1 (**E**). *P < 0.05, **P < 0.01, ***P < 0.001.

As described earlier, MCPIP1 recognizes a specific secondary RNA structure, stem-loop, to degrade target mRNAs, and the UAU or UGU loop sequence is the preferential target for MCPIP1^5^. Indeed, HBV epsilon has two UGU sequences within the loop structure (Fig. S4A). Thus, we hypothesized that MCPIP1 targeted the stem-loop structures of HBV RNA to reduce viral RNA. We constructed expression vectors of mutant pgRNA reporters lacking 5′-and/or 3′-epsilon structures (Fig. 3A). They were cotransfected with the expression vectors of GFP or GFP-tagged MCPIP1, and the NL activity was determined (Fig. 3B,C). The result revealed that the RNA levels of 5′-deleted or 3′-deleted epsilon pgRNA reporter were significantly less affected by MCPIP1 overexpression, compared to the wild type pgRNA. Deleting the other additively attenuated the effect, suggesting that MCPIP1 targets both of the epsilon structures to downregulate viral RNA.

Huh7 cells were cotransfected with the expression vector for MCPIP1 and pgRNA reporter vector to verify whether MCPIP1 binds to viral RNA. We found that FLAG-MCPIP1 immunoprecipitation enriched the viral RNA, but not the host HPRT, significantly more than FLAG-GFP, suggesting that MCPIP1 binds with HBV RNA (Fig. 3D,E). Furthermore, an in vitro cleavage assay was performed, as previously done for IL-6 mRNA and a precursor miRNA (pre-miRNA), to demonstrate that their stem-loop structures were targeted by MCPIP1^15,17^. We found that the recombinant MCPIP1 cleaved HBV epsilon RNA in vitro (Fig. S4B). Overall, these results suggested that MCPIP1 reduced HBV RNA via cleavage of the epsilon structures.

### IL-1β adopts MCPIP1 for viral RNA downregulation

**Figure 4:**
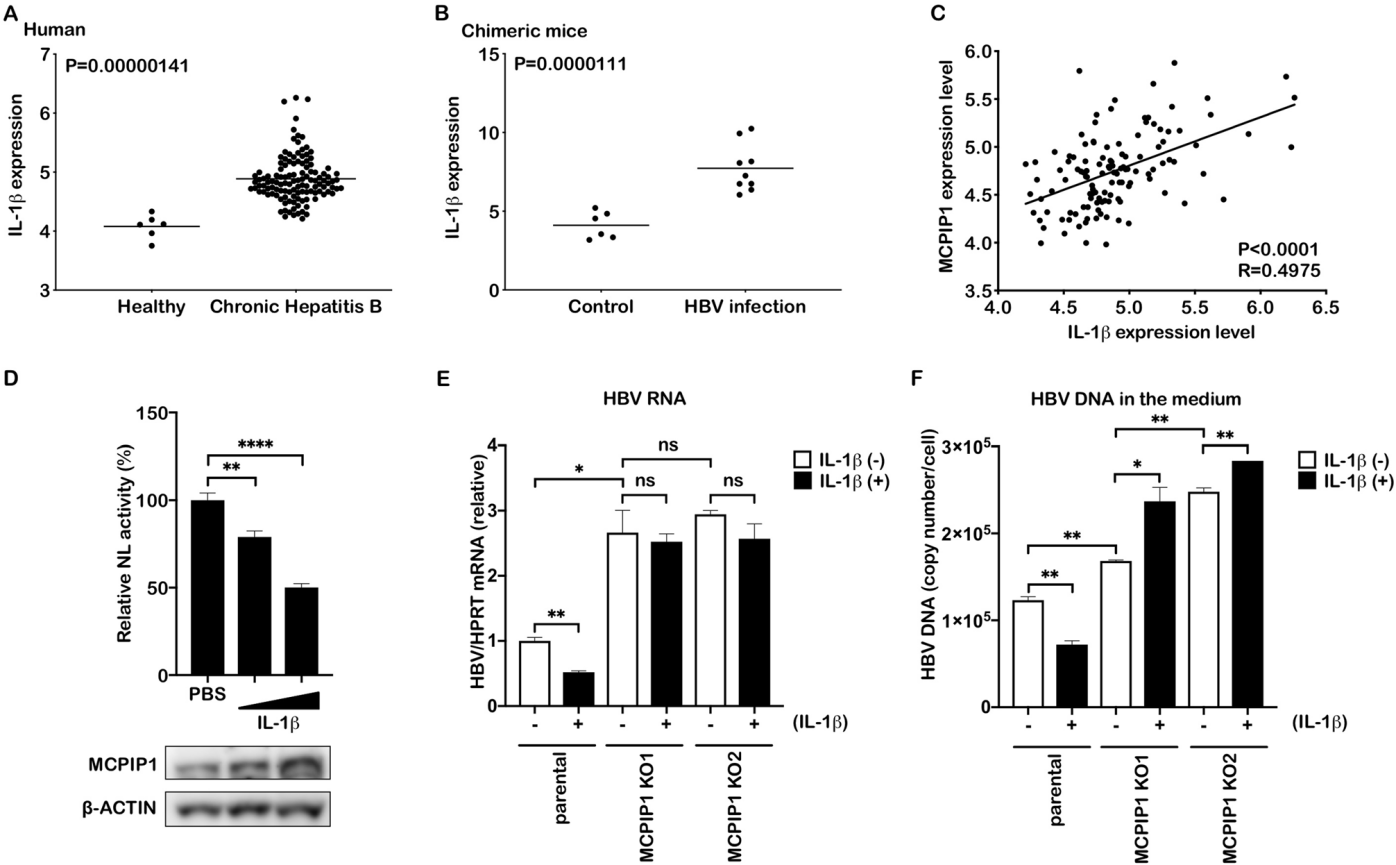
IL-1β adopts MCPIP1 for viral RNA downregulation. (**A**) IL-1β mRNA level in CHB (n = 122) and normal liver (n = 6, GSE83148). (**B**) IL-1β mRNA level in human liver-chimeric mice with 8 weeks after HBV infection (n = 9) and controls (n = 6, GSE52752). (**C**) Correlation between the mRNA levels of MCPIP1 and IL-1β in CHB patients. (**D**) Huh7 cells were cotransfected with pCMV1.2xHBV/NL, pCAG-SEAP, and pcDNA-CP, and cultivated for 24 h in the absence (PBS) or presence of IL-1β (100 and 200 ng/ml). The total lysates were subjected to luciferase assay and Western blotting. (E, F) Parental or MCPIP1 knockout Hep38.7-Tet cells were treated with or without 100 ng/ml IL-1β in the absence of tetracycline for 5 days. (**E**) RT-qPCR analysis to quantify HBV RNA level, normalized by HPRT. The result is indicated by the ratio to the untreated parental cells. (**F**) qPCR analysis to quantify HBV DNA in the culture supernatant. The result is presented as absolute copy numbers. *P < 0.05, **P < 0.01, ****P < 0.0001.

MCPIP1 is reportedly involved in immune response, such as macrophage activation, regulation of the inflammatory response, and antiviral activity, by cleaving target RNA such as IL-6 mRNA and pre-miRNA^2,4^, and is induced by proinflammatory cytokines, such as IL-1β, TNFα, and MCP-1^18–20^. We found that the expression of IL-1β was higher in the CHB livers than in the healthy controls, and HBV-infected human liver-chimeric mice than those in the control group (Fig. 4A,B). Moreover, IL-1β expression was positively correlated with MCPIP1 expression in HBV-infected patients (Fig. 4C). As we previously reported, IL-1β reduced pgRNA reporter activity in Huh7 cells^21^. And we found that MCPIP1 protein was also increased (Fig. 4D), and this led us to assess the contribution of MCPIP1 to the IL-1β-mediated anti-HBV activity in hepatocytes. The parental and MCPIP1 knockout Hep38.7-Tet cells were cultured in the presence of IL-1β. Treatment with IL-1β significantly reduced HBV RNA as well as culture supernatant HBV DNA in the parental cells (Fig. 4E,F). In contrast, IL-1β mediated reduction was not observed in the MCPIP1-knockout cells. For further verification, NTCP-overexpressing HepG2 cells^22^ were transfected with siMCPIP1, infected with HBV, and treated with IL-1β (Fig. S5A). In the absence of IL-1β, the viral RNA level was higher in the siMCPIP1 transfectants, compared to the control siRNA (Fig. S5B), as we found in the MCPIP1 knockout Hep38.7-Tet cells (Fig. 1F). Unexpectedly, IL-1β rather increased the viral RNA level in the siMCPIP1-transfected cells, while it decreased in the parental cells (Fig. S5B). Consistently, IL-1β treatment increased the viral RNA level in the MCPIP1-knocked out HepG2-NTCP cells, infected with HBV (Fig. S5C). The cellular DNA level was comparable between the IL-1β-treated and untreated cells. And when the knock-out HepG2-NTCP cells were transfected with the expression plasmid for MCPIP1, the wild type but not D141N mutant decreased the viral RNA and protein, compared to the mock transfectant (Fig. S5D-H). Taken together, these results suggest that MCPIP1 exerts an antiviral effect downstream of IL-1β.

## Discussion

In this study, we demonstrated the antiviral role of MCPIP1 that negatively regulates HBV RNA level in viral replicating hepatocytes. MCPIP1 expression was upregulated in liver tissue with CHB and HBV-infected human liver-chimeric mice (Figs. S1A and S1B). Overexpression of MCPIP1 decreased HBV RNA both in vitro and in vivo (Figs. 1A,C, and S5E), and loss of endogenous MCPIP1 increased viral RNA in Hep38.7-Tet cells and HBV-infected HepG2-NTCP cells (Fig. 1F, S5B, and S5C). Its RNase activity, CCCH-type zinc-finger domain, and a proline-rich domain were shown to be important for its anti-HBV activity (Fig. 2). Both the 5′- and 3′- epsilon structures of HBV pgRNA were required for MCPIP1-mediated viral RNA reduction, and MCPIP1 can bind and cleave the viral epsilon structure (Fig. 3E, and S4). Moreover, MCPIP1 ablation attenuated IL-1β-mediated downregulation of HBV RNA (Figs. 4E, S5B and S5C). We previously showed that IL-1β decreased HBV RNA and further proposed that Activation-Induced cytidine Deaminase (AID) was one of the effector molecules for IL-1β-mediated downregulation of HBV RNA^21^. The results of this study suggest that MCPIP1 is another host factor that plays a role in IL-1β-mediated antiviral pathway.

All members of the MCPIP family possess an NYN and CCCH-type zinc-finger domain, which function in RNase and RNA-binding activities, respectively^2^. However, only MCPIP1 exhibited obvious anti-HBV activity (Fig. 1A), consistent with that against HIV, JEV and DEN^6,8^. The proline-rich C terminus, unique to MCPIP1 but not others, is reportedly important for its oligomerization and degradation of JEV RNA, DEN RNA, and pre-miRNA^6^. We found that the C-terminus deletion mutant, lacking the proline-rich domain of MCPIP1 454–599, abolished the inhibitory effects on HBV RNA (Fig. 2C). Thus, it is intriguing to speculate, yet remains to be verified, that oligomerization via the proline-rich C-termini explains anti-HBV activity, unique to MCPIP1, but not other MCPIPs.

We demonstrated that MCPIP1 could decrease HBV RNA in Hep38.7-Tet cells, even when the transcription was inhibited by addition of tetracycline (Fig. S3). Taken together with the in-vitro data that MCPIP1 can cleave HBV epsilon RNA (Fig. S4), we prefer the possibility that MCPIP1 directly degrades viral RNA. MCPIP1 recognizes stem-loop structures to degrade target RNAs, preferentially those containing UAU or UGU^5^. In line with this, the 5′- and 3′-epsilon structures of viral pgRNA have UGU loop structure (Fig. S4A) and are required for efficient MCPIP1 mediated viral RNA reduction (Fig. 3). While contribution of 5′-epsilon is slightly greater than that of 3′-epsilon to the MCPIP1-mediated HBV-RNA degradation (Fig. 3C), MCPIP1 could decrease HBsAg in the Hep38.7-Tet and HepG2-NTCP cells (Figs. 1H and S5F), suggesting that MCPIP1 can also target HBs mRNA, containing only 3′-epsilon. Meanwhile, Imam et al. proposed that 5′- and 3′-stem-loop structures play distinct roles in reverse transcription and stabilizing viral RNA^23^. Further studies are warranted to precisely determine the different property of 5′- and 3′- epsilon structures, as the substrate of MCPIP1. In addition, MCPIP1-mediated viral RNA reduction could be both epsilon-structure dependent and independent because MCPIP1-mediated viral RNA reduction is still slightly observed in pgRNA even without any epsilon structure (Fig. 3C). Consistent with this idea, Wilamowski et al.^17^ reported ribonuclease activity against single-stranded RNAs in MCPIP1. Furthermore, we cannot deny the possibility that MCPIP1 affects transcription of HBV genes, and not in a mutually exclusive manner, expression of other host factors as we reported^15,21,24^, regulating viral transcription or RNA stability.

IL-1β is upregulated in HBV-infected patients (Fig. 4A), and IL-1β polymorphism is associated with HBV infection^25,26^. Furthermore, HBe antigen suppresses IL-1β production by Kupffer cells^27^, implying that the IL-1β/MCPIP1 axis plays an antiviral role in the HBV-infected liver. MCPIP1 expression was upregulated in the HBV-infected liver (Fig. S1A), and positively correlated with IL-1β (Fig. 4C). Furthermore, IL-1β increased MCPIP1 in Huh7 cells (Fig. 4D), as reported by other groups^18–20^. Meanwhile, MCPIP1 reportedly regulates the mRNA level of IL-1β and MCPIP1 itself^28^, likely forming a negative feedback loop with IL-1β. Further studies are required to clarify the dynamics of anti-HBV activity by IL-1β-MCPIP1 axis in vivo, in human liver tissues.

IL-1β treatment increased the viral RNA level in the MCPIP1-depleted HepG2-NTCP cells (Figs. S5B and S5C), but not the Hep38.7-Tet cells (Fig. 4E). Considering that the viral RNA level is also affected by the upstream life cycle such as viral entry, cccDNA formation and transcription in the HepG2-NTCP cells, it is intriguing yet remains to be verified, that MCPIP1 also negatively regulates these steps, apart from the direct viral RNA degradation, by targeting proviral host mRNA and microRNA as we and other groups have reported^2,15^. In addition, we previously reported that IL-1β upregulates AID^21^, which degrades HBV RNA by recruiting the RNA exosome complex^24^. Up to now, we have proven that both MCPIP1 and AID can be induced by IL-1β to degrade HBV RNA through different mechanisms, and the exact association between these mechanisms and the contribution of each to anti-HBV activity by IL-1β remains to be determined.

In summary, we demonstrated that MCPIP1 could decrease HBV RNA level, most likely by cleaving viral RNA, and mediates antiviral effect by IL-1β. Further studies are necessary for clarifying the overall portrait of immunity against HBV propagation, involving IL-1β and MCPIP1.

## Methods

### Database

Gene expression profiles of CHB patients (GSE83148)^29^, HBV-infected human liver-chimeric mice (GSE52752), and PHHs (GSE69590)^10^ were downloaded from the Gene Expression Omnibus (GEO) public database. The expression level of MCPIP family and IL-1β was extracted by GEO2R.

### Plasmids

Expression vectors for MCPIP family proteins, including mutants (D141N and C306R) were described previously^15^. MCPIP1, 4 open reading frames were subcloned from FLAG-MCPIP expression vectors, into pGFP2-C2 vector (Packard Bioscience). Further, pCMV1.2xHBV/NL and their mutants, as well as the helper plasmids were described previously^11^. The MCPIP1 C-terminal truncated mutant (1–453) was obtained from Addgene. The MCPIP1 mutant C157A^16^ was generated from a FLAG-MCPIP1 wild type construct by Mutagenesis Basal Kit (Takara), and primers sequences are listed in Table S1.

### Cell culture, transfection, and reporter assay

Huh7 and 293FT cells were obtained from JCRB Cell Bank and Invitrogen, respectively, and cultured as described previously^11^. Hep38.7-Tet and HepG2-NTCP (C4) cells were established and maintained as described^14,22^. Briefly, Hep38.7 cells were established by subcloning HepAD38 cells (obtained from Dr. Christoph Seeger at Fox Chase Cancer Center, Philadelphia); and HepG2-NTCP (C4) cells by transfecting an expression plasmid for human NTCP into HepG2 cells, followed by neomycin selection. Plasmids were transfected into cells using Fugene 6 (Promega) or Lipofectamine 3000 (Thermo Fisher Scientific), according to the manufacturer’s instructions. Luciferase and SEAP activities were measured using the Nano-Glo Luciferase Assay Kit (Promega), and the SEAP Reporter Gene Assay Kit (Roche), respectively, according to the manufacturer’s protocol. IL-1β was obtained from Wako.

### HBV infection

HBV infection was performed as described previously^21,30^. HBV (genotype D) was prepared from the culture supernatant of Hep38.7-Tet cells. HepG2-NTCP cells were infected with HBV (6000–8000 GEq/cell) in the presence of 4% PEG8000. At 16 h post-infection, the infected cells were washed three times with medium and switched to fresh medium. The cells and culture supernatants were collected at 13 days post- infection.

### Mice

Hydrodynamics-based gene delivery was performed as described previously^11,13,31^. The animal experiment was approved by the ethical committee of the National Institute of Infectious Diseases, and conducted in accordance with its guideline regarding the care and use of laboratory animals.

### Western blot analysis

Western blot analysis was performed as previously described^11,24,32^. Briefly, cells or homogenized liver samples were lysed with lysis buffer (62.5 mM tris at pH 6.8, 3% SDS, protease inhibitor [Roche, 05056489001]). Lysates were loaded onto an SDS polyacrylamide gel and transferred onto PVDF membranes (Bio-Rad) according to the manufacturer’s instructions. The membranes were blocked by tris-buffered saline containing 0.05% Tween20 and 5% skim milk. The primary antibodies used were as follows; anti-MCPIP1 (GeneTex, GTX110807); anti-GFP (Santa Cruz, sc-9996); anti-FLAG (Sigma, F7425); and anti-β-Actin (Sigma, A5316). Regarding the secondary antibody, anti-mouse (CST, 7076) and anti-rabbit (CST, 7074) IgG HRP-linked antibodies were used. Signals were developed by EzWestLumi plus (ATTO) and visualized by Amersham Imager 680.

### qPCR

Culture supernatant DNA was extracted using a NucleoSpin kit (Takara). Total RNA was extracted using RNeasy Mini Kit (QIAGEN), treated with amplification grade DNase I (Thermo Fisher Scientific), and then reverse transcribed into cDNA with a High Capacity cDNA Reverse Transcription Kit (ABI), according to the manufacturer’s instructions. qPCR analysis was performed using TB Green Premix Ex Taq II (Takara) with StepOnePlus Real-Time PCR systems (ABI). Primers sequences are listed in Table S1. Expression of HBV RNA mRNA was normalized to that of the endogenous HPRT. An HBV replicon plasmid was used as a standard to absolutely quantify HBV copy numbers in the culture supernatant.

### RNA immunoprecipitation

RNA immunoprecipitation (RIP) was performed as previously described^24^. Briefly, Huh7 cells were transfected with the HBV-NL and pcDNA-CP vector together with those for either FLAG-GFP (mock) or FLAG-MCPIP1. The transfected cells were lysed with phosphate-buffered saline containing 0.1% Tween 20, 1% triton-X, 1-mM EDTA, protease inhibitor (Roche, 05,056,489,001), and 2% glycerol. After centrifugation, crude lysates were equilibrated with anti-FLAG M2 agarose beads for 4 h. The immune complexes collected were washed in lysis buffer 10 times and were then washed in lysis buffer containing an additional 100 mM NaCl. FLAG-MCPIP1 and RNA complexes were eluted using free 3xFLAG peptides (Sigma, F4799). The immunoprecipitation efficiency was confirmed by Western blotting. HBV and HPRT RNA in crude lysates and the eluted fraction from mock and FLAG-MCPIP1 transfectants were quantified by RT-qPCR.

### In vitro RNA cleavage assay

The in vitro RNA cleavage assay analysis was performed as described previously^17^. The reaction mixture containing 7.5 µM 5′-FAM conjugated HBV epsilon RNA (Table S1) and 2 µM recombinant MCPIP1 (ORIGENE, TP301381) was incubated at 37 °C for 4 h in 25 mM tris–HCl at pH 7.9, 150 mM NaCl, 10% Glycerol, 2.5 mM MgCl_2_, 1 mM DTT, 0.5 mM EDTA, and 0.05 mM ZnCl_2_. The reaction was terminated by freezing in liquid nitrogen. The samples were then mixed with 2 × RNA Loading Buffer with ethidium bromide (Wako, 185-02561), denatured at 70 °C 10 min, chilled on ice, and loaded on 15% SuperSepTMRNA (Wako, 194-15881). Fluorescence was detected using LAS 4000.

### CRISPR/Cas9-mediated gene targeting

MCPIP1 knockout cells were established as described previously^32^. The MCPIP1 CRISPR plasmids (Santa Cruz, sc-401790-NIC, sc-40190-NIC-2) were transfected into Hep38.7-Tet cells. The oligonucleotides inserted into pX330-U6-Chimeric_BB-CBh-hSpCas9 (Addgene plasmid # 42230), targets MCPIP1 exon 2 (5′-GTGGACTTCTTCCGGAAGCT-3′). The vector was co-transfected into HepG2-NTCP cells with an expression vector of puromycin-resistant gene pIRES.Puro.EGFP (Addgene plasmid # 24176). The cells were cloned by limiting dilution, after selection by puromycin.

### Quantification of HBsAg and HBeAg

HBsAg and HBeAg levels in the culture media were measured by Lumipulse G1200 (Fujirebio), using a chemiluminescent enzyme immunoassay (Fujirebio, 296851, 231517) as per the manufacturer’s protocol.

### Immunofluorescence analysis

Immunofluorescence analysis was performed as described previously^33^. HepG2-NTCP knockout cells were transfected with expression vectors of GFP-Mock, -MCPIP1 or -D141N mutant 2 days before HBV infection, as well as day 4 and 10 post infection. The cells were harvested at day 13 and fixed with 4% paraformaldehyde, permeabilized with 0.3% Triton X-100, and incubated with Anti-HBc antibody (Thermo Fisher Scientific, RB1413A) and donkey anti-rabbit IgG (H + L) conjugated to Alexa 594 as primary and secondary antibodies, respectively.

### siRNA transfection

Stealth-grade siRNAs for human MCPIP1 and control (GAPDH) were purchased from Invitrogen. Lipofectamine RNAiMAX was used to perform siRNAs transfections according to the manufacturer’s instructions.

### Statistics

Statistical analysis was performed using GraphPad Prism. Significance between two groups was determined using Student’s t-test. Linear regression was employed to determine correlation between mRNA expressions. Pearson’s correlation analysis was conducted to investigate correlation between variables. P-values < 0.05 were considered statistically significant.

## Supplementary information

Supplementary Information 1.
