# Supplementary material for: MCPIP1 reduces HBV-RNA by targeting its epsilon structure
Source: Sci Rep. 2020 Nov 27;10:20763. doi: 10.1038/s41598-020-77166-z (PMC7699622; doi:10.1038/s41598-020-77166-z)

Supplementary information

MCPIP1 reduces HBV-RNA by targeting its epsilon structure

Yingfang Li [a, b], Lusheng Que [a, b], Kento Fukano [b], Miki Koura [a], Kouichi Kitamura [a, b], Xin Zheng [b], Takanobu Kato [b], Hussein Hassan Aly [b], Koichi Watashi [b], Senko Tsukuda [b], Hideki Aizaki [b], Noriyuki Watanabe [b], Yuko Sato [c], Tadaki Suzuki [c], Hiroshi I. Suzuki [d, e], Kazuyoshi Hosomichi [f], Makoto Kurachi [a], Kousho Wakae [a, b]*, Masamichi Muramatsu [a, b]*.

[a] Department of Molecular Genetics, Graduate School of Medical Science, Kanazawa University, Kanazawa, Ishikawa 920-8640, Japan, [b] Department of Virology II, National Institute of Infectious Diseases, Tokyo, 162-8640, Japan, [c] Department of Pathology, National Institute of Infectious Diseases, Tokyo, 162-8640, Japan, [d] David H. Koch Institute for Integrative Cancer Research, Massachusetts Institute of Technology, Cambridge, MA 02139, USA, [e] Division of Molecular Oncology, Center for Neurological Diseases and Cancer, Nagoya University Graduate School of Medicine, Nagoya, 466-8550, Japan, [f] Department of Bioinformatics and Genomics, Graduate School of Advanced Preventive Medical Sciences, Kanazawa University, Kanazawa, Ishikawa 920-8640, Japan

***To whom correspondence should be addressed:**

Masamichi Muramatsu and Kousho Wakae

Department of Virology II, National Institute of Infectious Diseases

1-23-1 Toyama, Shinjuku-ku, Tokyo 162-8640, Japan.

Email: muramatsu@nih.go.jp and wakae@nih.go.jp

Tel: +81-3-5285-1111

FAX: +81-3-5285-1161

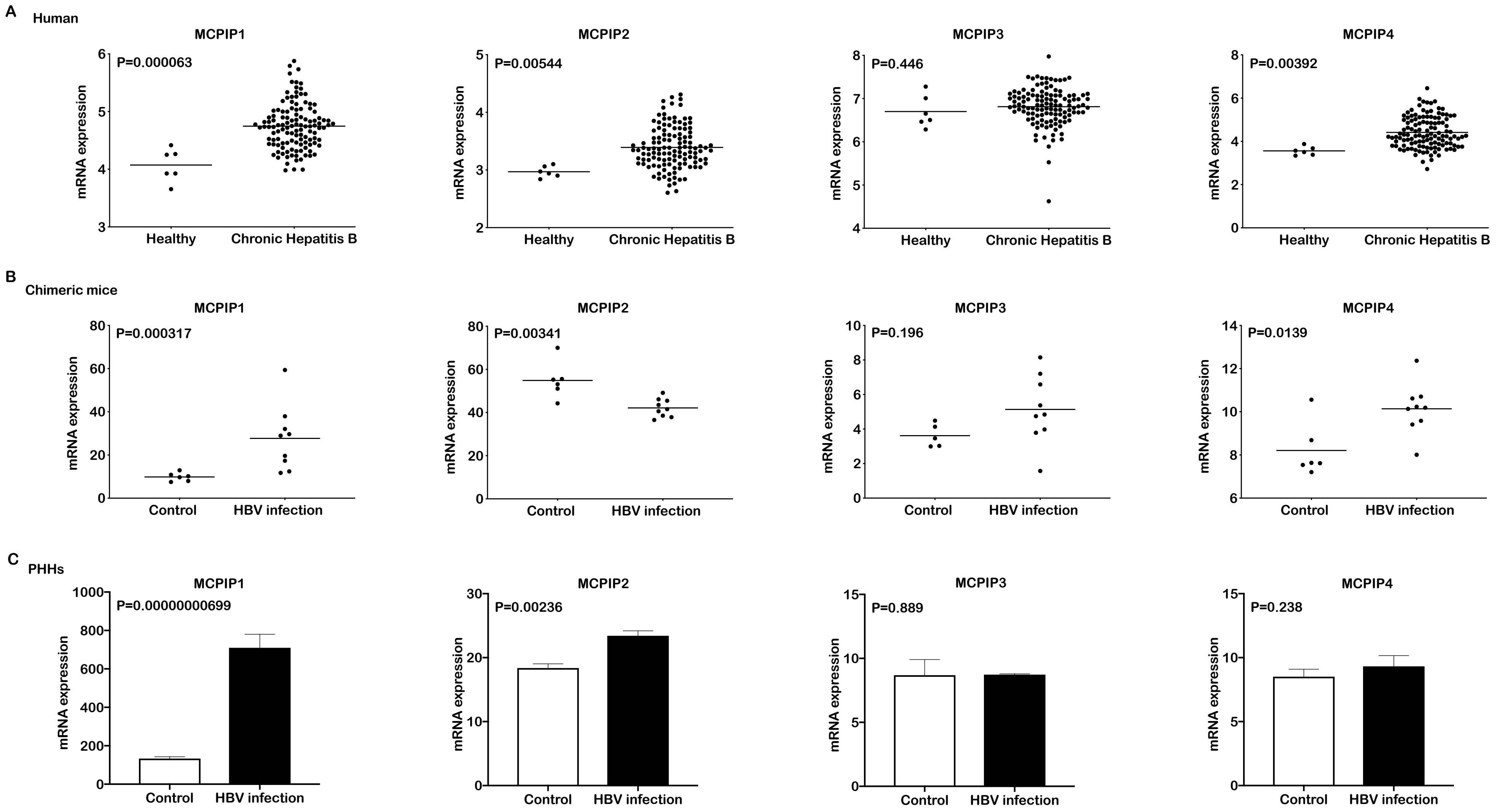

Figure S1. mRNA of MCPIPs expression in HBV-infected liver

(A, B, C) mRNA levels of MCPIP family in liver tissues from patients with chronic hepatitis B (n = 122) and healthy controls (n = 6) (A, GSE83148), in human liver-chimeric mice with 8 weeks after HBV infection (n = 9) and controls (n=6) (B, GSE52752), and in HBV-infected PHHs (n = 3) and control (n=3) (C, GSE69590).

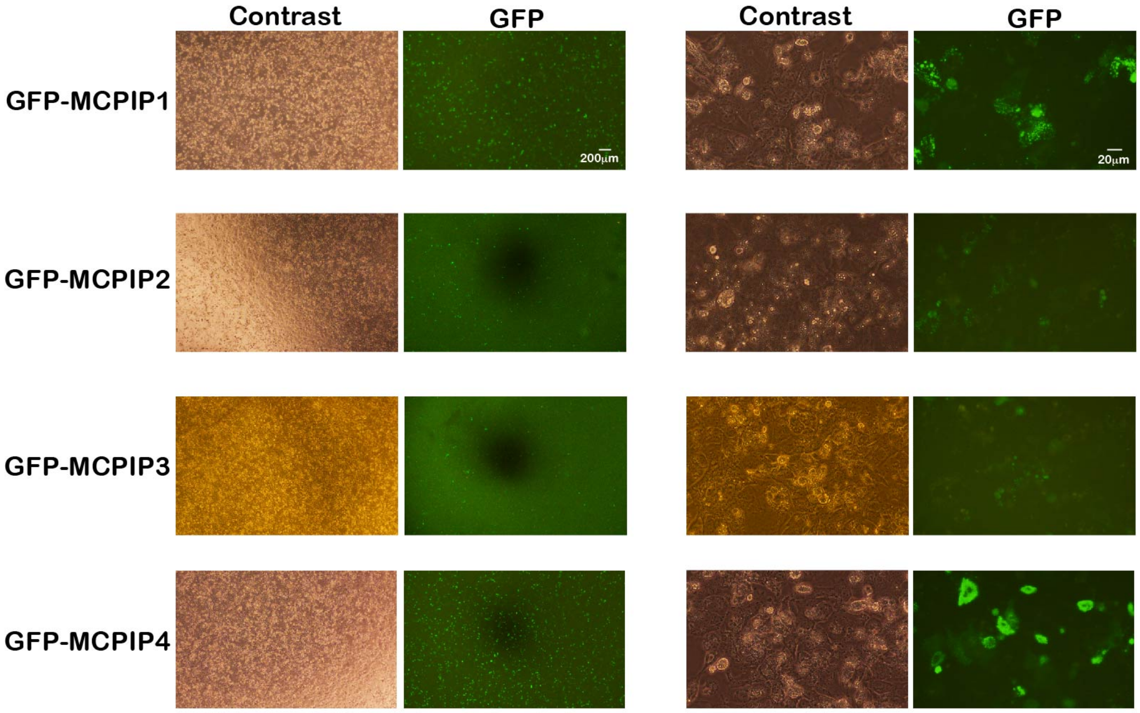

Figure S2. Transfection efficiency of MCPIPs

Huh7 cells were transfected with pgRNA reporter (pCMV1.2xHBV/NL), helper plasmid (pcDNA-CP), and pCAG-SEAP, together with GFP or GFP-MCPIP expression vectors. The cells were examined with a fluorescence microscope 24 hours post-transfection.

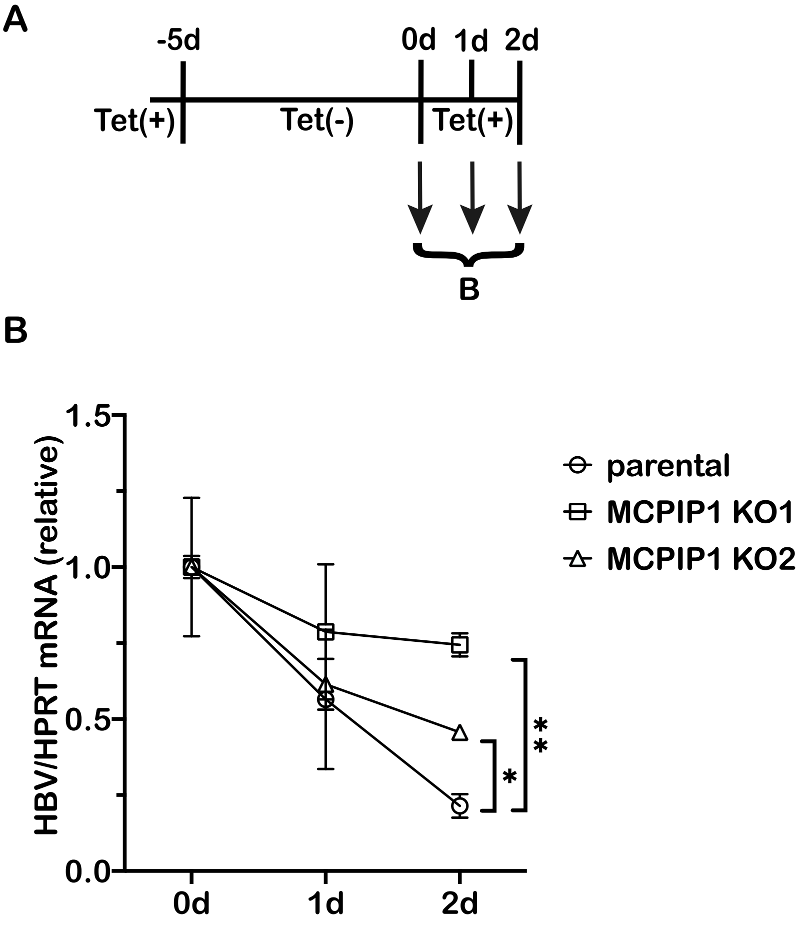

Figure S3. MCPIP1 destabilized HBV RNA

(A) Parental or MCPIP1 knockout Hep38.7-Tet cells were cultured with or without tetracycline (for 2 and 5 days, respectively). (B) Total RNA was harvested at the indicated time points in A (0, 1, 2 days) and subjected to RT-qPCR analysis to determine HBV RNA level, normalized by HPRT. * P<0.05, ** P<0.01.

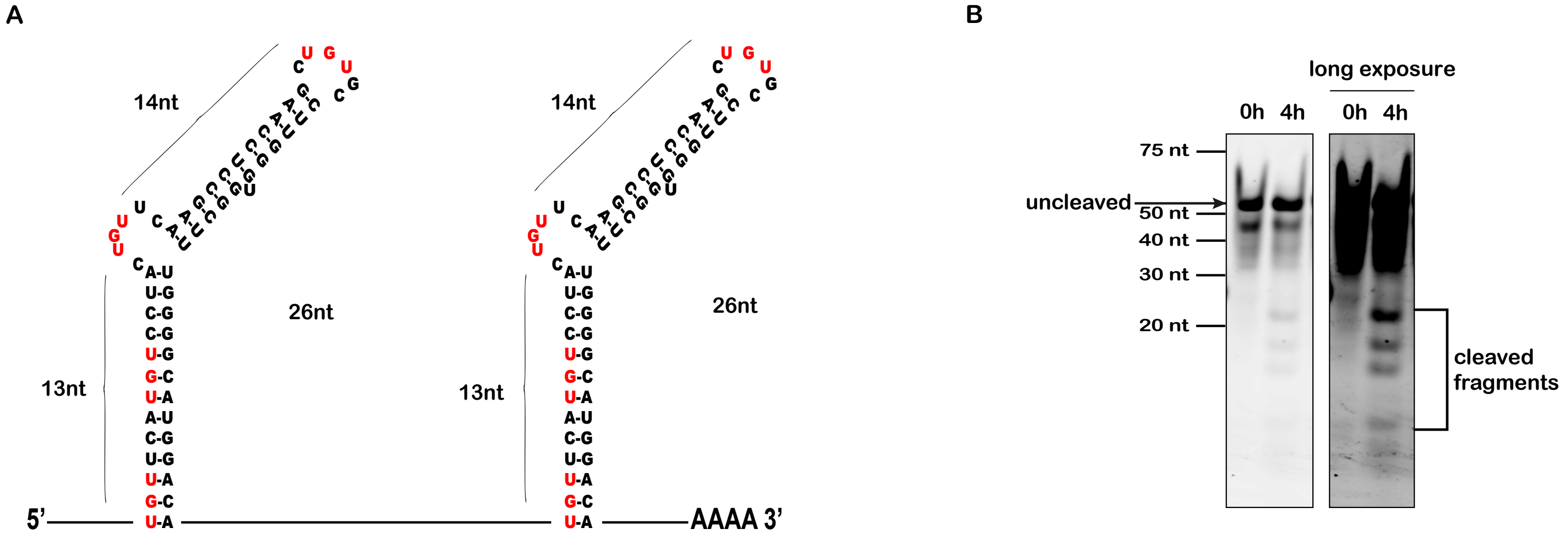

Figure S4. Structure of HBV pgRNA and in vitro cleavage assay of HBV epsilon RNA

(A) Schematic stem-loop structure of HBV epsilon. UGU loop sequence is marked in red. (B) 5'-FAM- conjugated HBV epsilon RNA (61nt) was incubated with recombinant MCPIP1 for the indicated times (0h or 4h), and run on a 15% denaturing urea polyacrylamide gel. The ticks in the figure indicate the position of molecular-weight markers.

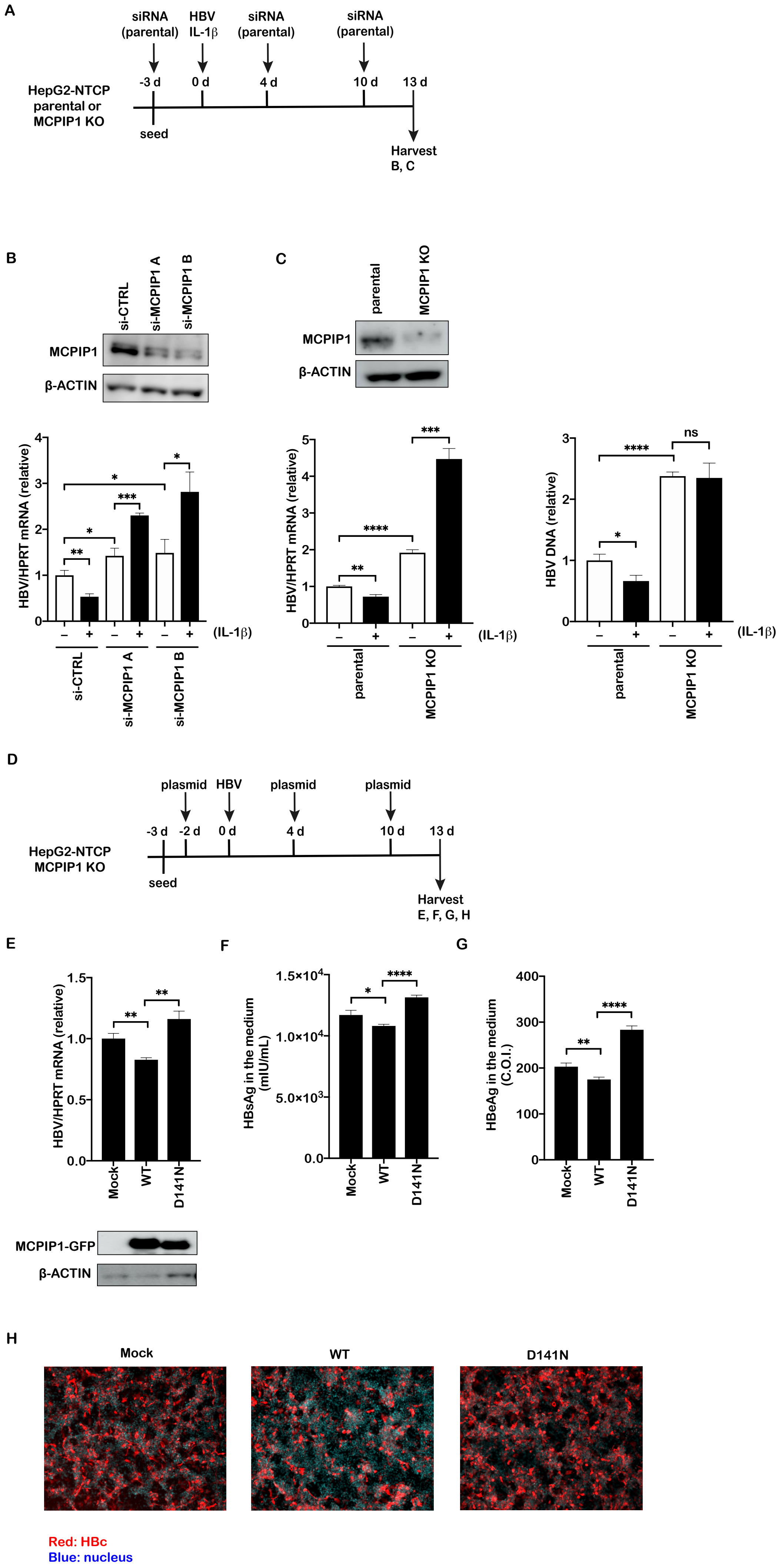

A
siRNA (parental)
HBV IL-1β
siRNA (parental)
siRNA (parental)
-3 d
0 d
4 d
10 d
13 d
HepG2-NTCP parental or MCPIP1 KO
seed
Harvest B, C
B
si-CTRL
si-MCPIP1 A
si-MCPIP1 B
MCPIP1
β-ACTIN
HBV/HPRT mRNA (relative)
(IL-1β)
C
parental
MCPIP1 KO
MCPIP1
β-ACTIN
HBV/HPRT mRNA (relative)
HBV DNA (relative)
ns
(IL-1β)
D
plasmid
HBV
plasmid
plasmid
-3 d
-2 d
0 d
4 d
10 d
13 d
HepG2-NTCP MCPIP1 KO
seed
Harvest E, F, G, H
E
HBV/HPRT mRNA (relative)
Mock
WT
D141N
MCPIP1-GFP
β-ACTIN
F
HBsAg in the medium (mIU/mL)
1.5×10^4
1.0×10^4
5.0×10^3
0.0
G
HBeAg in the medium (C.O.I.)
H
Mock
WT
D141N
Red: HBc
Blue: nucleus

Figure S5. IL-1β adopts MCPIP1 for its viral RNA reduction in the HBV-infected HepG2-NTCP cells.

(A-C) HepG2-NTCP cells (parental or MCPIP1 KO) were transfected with siRNA (si-MCPIP1 or control siRNA, for B) for indicated time and infected with HBV in the presence or absence of IL-1β (100ng/mL). Cells were harvested at 13 days post-infection. (B) (Upper) The protein level of MCPIP1 was determined by Western blotting. (Lower) RT-qPCR analysis to determine the viral RNA level, normalized by HPRT. (C) (Upper) MCPIP1 expression was confirmed by Western blotting. (Lower) Parental or MCPIP1 knockout HepG2-NTCP cells were infected with HBV, and cultivated in the presence or absence of IL-1β for 13 days. Cells were harvested and subjected to RT-qPCR (left) and DNA-qPCR (right) analysis to determine cellular viral RNA and DNA level, respectively. (D-H) MCPIP1 knockout HepG2-NTCP cells were transfected with mock or GFP-MCPIP1(WT or D141N) expression vectors 2 days before infection, as well as day 4 and day 10 post-infection. At 13 days after HBV infection, the cells were subjected to RT-qPCR (E, normalized by HPRT), chemiluminescent enzyme immunoassay to quantify HBsAg and HBeAg levels in the culture supernatant (F, G, expressed as international units mIU/ml and Cut-Off-Index (C.O.I.), respectively), and immunofluorescent staining of HBc protein (H, HBc: *red,* counterstained by DAPI). * $P<0.05$, ** $P<0.01$, *** $P<0.001$, **** $P<0.0001$.

Supplementary Table S1: Primers used in this study

assay	Figures	target genes	orientation	sequence	reference
qPCR	Figs. 1F, 1G, 4E, 4F, S5B, S5C, S5D and S5E	HBV (2090-2210, the red arrows indicate the position of the primers, below)	F	GAATTGATGACTCTAGCTACCTG	Kitamura et al. PLoS. Pathog. 2011<br>Liang et al. PLoS. Pathog. 2015
			R	GAAACCACAATAGTTGCCTGATC	
		HPRT	F	GCCCTGGCGTCGTGATTAGT	
			R	CGAGCAAGACGTTCAGTCCTGTC	
RNA immunoprecipitation	Fig. 3E	HBV epsilon	F	CATGTCCCACTGTTCAAGCCT	this study
			R	AAGCCACCCAAGGCACAGCT	
In vitro RNA cleavage assay	Fig. S5	HBV epsilon RNA	5'FAM	UGUUCAUGUCCUACUGUUCAAGCCUCCAAGCUGUGCCUUGGGUGGCUUUGGGGCAUGGACA	this study
Contruction of MCPIP1 mutant C157A	Fig. 2D	MCPIP1	F	TTCTCCGCCCGGGGCATC	this study
			R	GCCCCGGGCGGAGAAGACCTC	

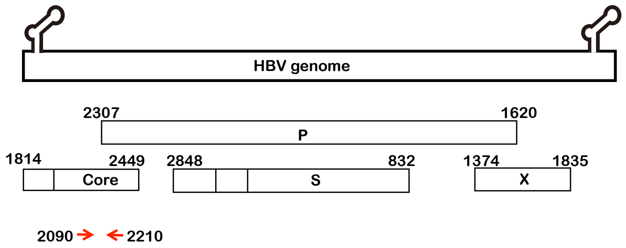

Original images

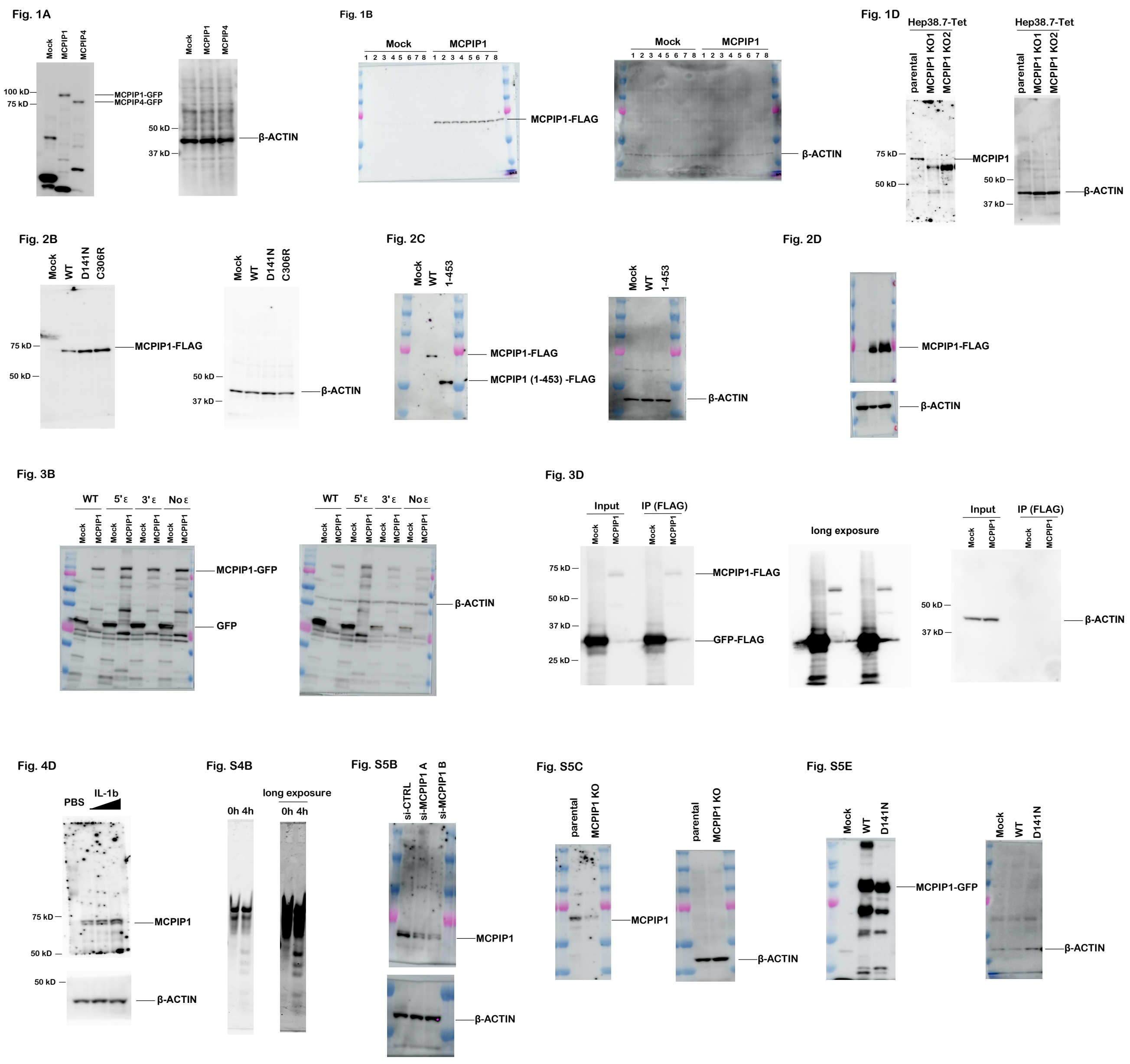

Supplement: Supplementary file 1 — Supplementary Information 1. [file 41598_2020_77166_MOESM1_ESM.pdf]
